# Supplementary material for: Causal Relationship between Aging and Anorexia Nervosa: A White-Matter-Microstructure-Mediated Mendelian Randomization Analysis
Source: Biomedicines. 2024 Aug 16;12(8):1874. doi: 10.3390/biomedicines12081874 (PMC11351342; doi:10.3390/biomedicines12081874)
Supplement: Supplementary file 1 [file biomedicines-12-01874-s001.zip › Supplementary Figure/Figure S4/Singe SNP plot of MR analysis for TL to ubm-b-1483.pdf]

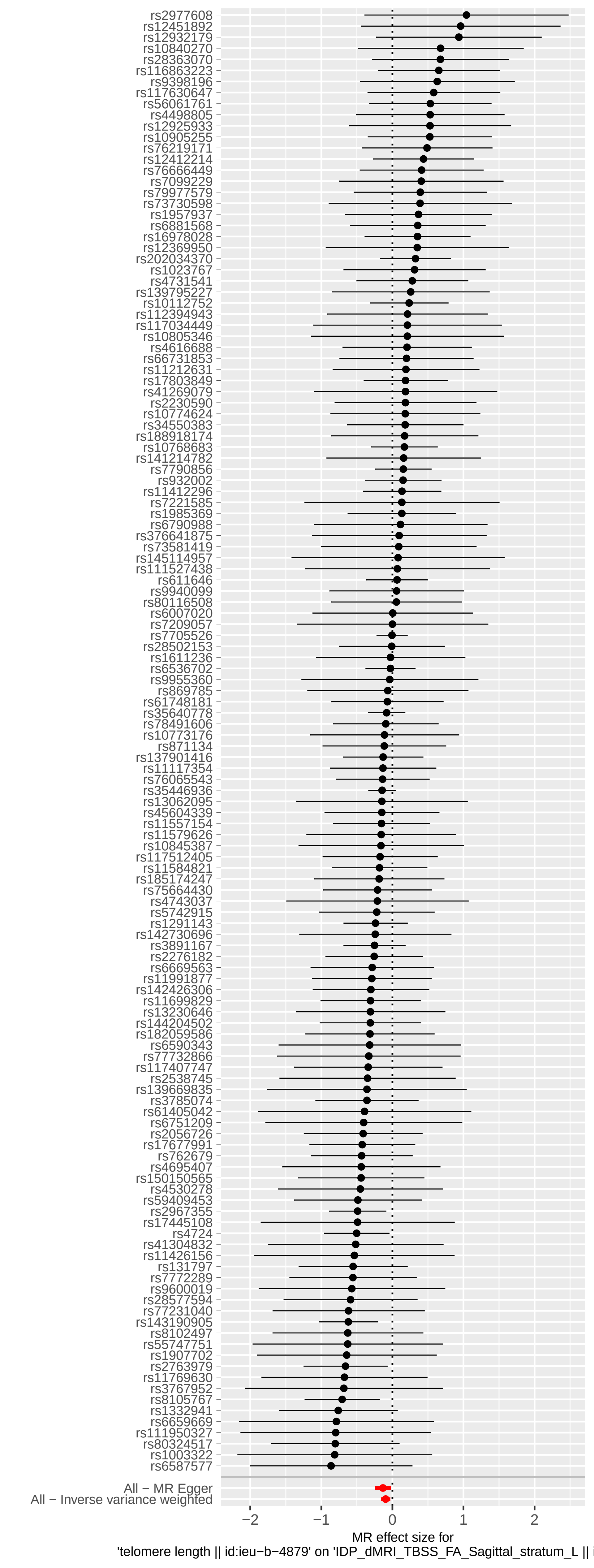

rs2977608
rs12451892
rs12932179
rs10840270
rs28363070
rs116863223
rs9398196
rs117630647
rs56061761
rs4498805
rs12925933
rs10905255
rs76219171
rs12412214
rs76666449
rs7099229
rs79977579
rs73730598
rs1957937
rs6881568
rs16978028
rs12369950
rs202034370
rs1023767
rs4731541
rs139795227
rs10112752
rs112394943
rs117034449
rs10805346
rs4616688
rs66731853
rs11212631
rs17803849
rs41269079
rs2230590
rs10774624
rs34550383
rs188918174
rs10768683
rs141214782
rs7790856
rs932002
rs11412296
rs7221585
rs1985369
rs6790988
rs376641875
rs73581419
rs145114957
rs111527438
rs611646
rs9940099
rs80116508
rs6007020
rs7209057
rs7705526
rs28502153
rs1611236
rs6536702
rs9955360
rs869785
rs61748181
rs35640778
rs78491606
rs10773176
rs871134
rs137901416
rs11117354
rs76065543
rs35446936
rs13062095
rs45604339
rs11557154
rs11579626
rs10845387
rs117512405
rs11584821
rs185174247
rs75664430
rs4743037
rs5742915
rs1291143
rs142730696
rs3891167
rs2276182
rs6669563
rs11991877
rs142426306
rs11699829
rs13230646
rs144204502
rs182059586
rs6590343
rs77732866
rs117407747
rs2538745
rs139669835
rs3785074
rs61405042
rs6751209
rs2056726
rs17677991
rs762679
rs4695407
rs150150565
rs4530278
rs59409453
rs2967355
rs17445108
rs4724
rs41304832
rs11426156
rs131797
rs7772289
rs9600019
rs28577594
rs77231040
rs143190905
rs8102497
rs55747751
rs1907702
rs2763979
rs11769630
rs3767952
rs8105767
rs1332941
rs6659669
rs111950327
rs80324517
rs1003322
rs6587577
All − MR Egger
All − Inverse variance weighted
−2
−1
0
1
2
MR effect size for
'telomere length || id:ieu−b−4879' on 'IDP_dMRI_TBSS_FA_Sagittal_stratum_L || i
